# Missingness in the expanded prostate cancer index short form (EPIC-26) – prevalence, patterns, and explanatory factors

**DOI:** 10.1186/s12955-023-02175-1

**Published:** 2023-08-14

**Authors:** Anna-Maija Talvitie, Mika Helminen, Hanna Ojala, Teuvo Tammela, Anssi Auvinen, Ilkka Pietilä

**Affiliations:** 1 Faculty of Social Sciences, Tampere University, Tampere, Finland; 2 Tays Research Services, Tampere University Hospital and Faculty of Social Sciences, Tampere University, Tampere, Finland; 3 Faculty of Medicine and Health Technology, Tampere University, Tampere, Finland; 4 Faculty of Social Sciences, University of Helsinki, Helsinki, Finland

**Keywords:** The Expanded Prostate Cancer Index Composite Short Form, Quality of life, Prostate cancer, Missing data, Validity, Patient-reported outcome measures

## Abstract

**Background:**

Researchers and clinicians using common clinical assessments need to attend to the prevalence of missing data to ensure the validity of the information gathered. The Expanded Prostate Cancer Index Short Form (EPIC-26) is a commonly used measurement scale used for assessing patients’ quality of life, but the measure lacks comprehensive analysis on missing data. We aimed to explore the quantity of missing answers in EPIC-26 and to characterize patterns and possible explanations of missing data in the survey.

**Methods:**

The survey sample consisted of 625 Finnish prostate cancer patients who participated in a study with a 1-year follow-up with three measurement points (0, 6, and 12 months). Descriptive statistics were used to describe the study population and missingness level. A logistic regression was performed for each EPIC domain to study factors related to missingness during the follow-up.

**Results:**

Proportions of missing answers in EPIC-26 were low (3.1–3.9%) between survey rounds. As much as 37% of patients left at least one question unanswered during their follow-up. The hormonal domain produced the most missing answers. Questions about breast tenderness/enlargement (question 13.b.), hot flashes (question 13.a.), frequency of erections (question 10.), and ability to reach orgasm (question 8.b.) were most frequently left unanswered. Higher age, lower education level, no relationship, more severe cancer, lower function scores in some EPIC domains, lower treatment satisfaction or self-rated health were associated with missingness.

**Conclusions:**

Questions 13.b. and 13.a. might be considered female-specific symptoms, thus difficult to comprehend unless patients had already experienced side effects from androgen deprivation therapy. Questions 10. and 8.b. might be difficult to answer if the patient has been sexually inactive. To improve the measure’s validity, the questionnaire’s hormonal section requires additional explanation that the inquired symptoms are common treatment side effects of anti-androgen therapy; questions 8–10 require a not-applicable category for sexually inactive patients.

**Supplementary Information:**

The online version contains supplementary material available at 10.1186/s12955-023-02175-1.

## Background

Patient-reported outcome measures (PROMs) have become firmly established in medicine as a routine part of developing and evaluating health care service delivery and quality of care [1]. PROMs are especially important in evaluating theoretical constructs or subjective characteristics that are not directly observable. Such characteristics, for example, health-related quality of life (HRQoL) or severity of symptoms, beliefs, and attitudes require carefully developed measurement scales to acquire robust estimations that can be used in clinical work or scientific research [2]. Thus, all factors affecting the validity of these scales must be considered carefully.

Missing or inconsistent responses on common clinical outcome measurements represent a challenge for researchers and clinicians because differential nonresponse can affect the conclusions that can be drawn from data [3]. Additionally, a high percentage of missing data reduces the representativeness of the selected sample, can cause bias and lead to a decrease in statistical power. It also negatively affects the validity and efficiency of the conducted analyses [4]. Missing data become an issue, especially when data are missing in a multi-item, health-related instrument that measures a latent construct that is difficult or impossible to measure directly.

### Definition of missing data

Questionnaire surveys typically contain missing or inconsistent data which, however, is not necessarily problematic [3]. Missing data may be classified according to the nature and degree of randomness with three categories: Missing Completely at Random (MCAR), Missing at Random (MAR), or Missing Not at Random (MNAR) [5]. If data are missing completely at random (MCAR), missingness cannot be accounted for by any variable. MCAR data are generally not a concern for statistical conclusion validity and can be ignored. However, data that are either MAR or MNAR can bias estimates from statistical analyses [3]. Data that are missing at random (MAR) may be related to other observed variables in the dataset but are independent of the true value of the missing data, because certain groups may not respond to a question due to an underlying reason [3, 5, 6]. If data are missing not at random (MNAR), missingness on a given item is directly related to the process influencing that variable and poses a threat to statistical conclusion validity [7]. Depending on the extent, type and form of missing data, researchers can employ common analysis techniques such as complete case analysis, maximum likelihood methods or imputation methods to account for the missingness [5].

The implications for bias in statistical analyses vary, so it is important for researchers and clinicians to consider rates and patterns of missingness in their data and report their findings [3]. Bannon (2015) [6] suggests that researchers should consider both *percentage of missing data values per study participant* and *percentage of study participants that have missing values* to decide reasonable methods for handling missing data when possible. Individuals with missing data might be systematically different from those with complete information, either regarding the outcome of interest or their prognosis. Identifying the profile of individuals with missing data is thus important for a study and its results to be valid [4].

### EPIC-26 as treatment evaluation tool

Several measurements have been developed to study different medical conditions and support clinical work. For prostate cancer patients, incidence and severity of treatment side effects are important measures of quality of life but are also crucial for treatment development and treatment quality evaluation. A suitable treatment option can be recommended to patients based on information on the probability of certain treatment side effects in certain treatment options.

Prostate cancer main treatment options are radiation therapy, surgery, hormonal treatment, and active surveillance. Common treatment side effects of radiation therapy include erectile dysfunction, urinary and bowel symptoms. Surgical removal of the prostate, prostatectomy, causes erectile disfunction and urinary leakage. Hormonal treatment options are usually followed by symptoms such as hot flashes, weight gain, breast growth, sexual changes, and depressiveness [8]. Active surveillance, which means monitoring a low-risk cancer with regular PSA testing, MRIs and biopsies, causes often anxiety, when patients have to live with a cancer diagnosis [8].

The Expanded Prostate Cancer Index (EPIC-50) [9] is a common quality of life (QoL) assessment instrument for prostate cancer patients. This disease-specific measure includes 50 Likert-scale questions considering five symptom domains and experienced bother of these symptoms: urinary incontinence, urinary irritation/obstruction, bowel, sexual, and hormonal symptoms indicating the common treatment side effects of surgery, radiation therapy, and hormonal therapy. EPIC-50 was developed from the UCLA Prostate Cancer Index (UCLA PCI) [10] that has 20 items covering both function and bother within the urinary, sexual and bowel domains but lacked hormonal items. The major changes in developing EPIC-50 were adding hormonal items and changing the balance between function and bother items to emphasize the experienced problems with treatment side effects [11].

EPIC-50 has been proven valid and reliable in multiple populations (e.g., [9, 12–14]), and patient compliance has been proven satisfactory [9]. An abbreviated version of EPIC (EPIC-26) [15] and a 16-item EPIC-CP for clinical practice [16] have also been developed. EPIC-26 was developed to facilitate HRQoL measures in a broader range of prostate cancer research and practice settings [15]. The survey includes the same five symptom domains as EPIC-50 but has only 26 items (see Appendix). EPIC-26 includes eight function items and 18 symptom-related bother items [11]. EPIC-CP is otherwise similar but lacks all conceptually overlapping items from EPIC-26 and is designed as a one-page tool that enables practitioners to calculate HRQoL scores at point of care [16].

These three EPIC questionnaires are frequently used globally, but only a few studies have reported their missing response rates. Szymanski et. al. (2010) [15] report in an EPIC-26 validation study that missing data were minimal within their data of 252 participants, with a median of 7 missing responses (2.8%) for the 26 items (range 2–14 [0.8%-5.6%]). To our knowledge, no studies on missing data exist on EPIC-50 nor is it commented on the validation study by Wei et. al. (2000) [9]. Chang et. al. (2011) [16] reported in their article on development and validation of the 16-item EPIC-CP that 89% of their cohort participants (*n* = 307) completed the questionnaire without any missing items. Brundage et al. (2019) [17] noted that sexual health item completion rates were among the lowest rates, ranging from 91–92% in their study evaluating EPIC-CP’s usefulness for ambulatory cancer care.

Our longitudinal research project, with three completed survey rounds with EPIC-26 for 625 Finnish prostate cancer patients, had 38% of the patients with missing responses on the first survey round measured at diagnosis time. Furthermore, it seemed that some EPIC domains produced more missing data than others. We have previously reported with qualitative data collected among participants of the project that a lack of suitable answer options increases missing data and produces differing answering strategies to some EPIC-26 questions [18]. Patients appeared to have difficulties especially with sexual items 8–10 because there are no suitable answer options for those who have not been sexually active or even desired to have an erection during the past four weeks. Patients with a urinary catheter faced difficulties in questions 1–4 [18]. Due to these challenges, and with no existing comprehensive analyses on missing data on EPIC measures, it is important to evaluate how these issues possibly affect the quality of statistical data received.

### Aim

This study’s aim is to explore the quantity of missing answers in the five EPIC-26 survey domains and to characterize patterns and explanations of missingness in a sample of 625 Finnish prostate cancer patients. The study aims to identify patients who struggle to give answers to certain questions, but in a broader spectrum it has a larger goal of promoting discussion regarding best practices in assessing prostate cancer patients’ HRQoL.

Our research questions were:

- 1. How much missing data are there in the different domains of EPIC-26 and how does missingness vary between measurement points?
- 2. What sociodemographic or clinical factors are associated with missingness?

## Methods

### Study design

This study is part of an ongoing multi-method research project (2017–2023) that combines a longitudinal survey and repeated interviews to examine patients’ QoL from a prostate cancer diagnosis to three years postdiagnosis. The research project was previously described by Talvitie et al. (2022) [18]. We recruited newly diagnosed prostate cancer patients from one hospital district in Finland. The patients received the first questionnaire, a study brochure, and a consent form by post at diagnosis time. Those who responded and gave their consent were sent the same questionnaire at 6-, 12-, and 36-months postdiagnosis. The monthly response rate was 62% on average. By the time this research was conducted, three first survey rounds in our longitudinal project were complete and thus used in this study. No interview data were used in this study.

### Data

In addition to EPIC-26 measure, our questionnaire included general QoL measures and sociodemographic items which we used for studying factors associated to missingness in EPIC-26. The items chosen to represent sociodemographic status were: relationship status, education level, and working life status. We used the self-rated health section from the SF-36 instrument as a generic QoL measure. The scale includes five questions on perceived overall health (scale 0–100) [19]. We also asked patients about treatment satisfaction in the follow-up rounds [20] and used the item in our analyses.

We collected clinical data representing prognostic factors of cancer such as PSA-level (prostate specific antigen), Gleason score, TNM stages from the hospital register, and information on cancer treatments to study their association to missingness in EPIC-26. We stratified prostate cancer into low, intermediate, high risk, and advanced disease groups based on the European Association of Urology (EAU) risk classification utilizing information on PSA, TNM stages and Gleason score [21].

### Analysis

Descriptive statistics were used to describe our study population and the missingness level. We used the Friedman test to examine if EPIC mean scores changed statistically significantly due to time (Table 3). All EPIC variables were non-normal. In EPIC-26, the item measuring overall urinary bother (question 5. on Table 5) is not included neither in the urinary incontinence nor urinary irritation subscales based on Wei et al.’s (2000) instructions [9]. Therefore, Tables 4 and 6 that explore missingness in each EPIC domain ignore the global urinary bother question. The Cochran Q test for three matched groups was used to assess whether the number of patients with missing answers changed statistically significantly during the follow-up (Table 6).

The main outcome variable examined in the study was missingness of data during one-year follow-up. We first calculated the total number of missing answers during the whole follow-up per EPIC domain. This continuous variable was then dichotomized [complete data/one or more missing values (reference group)] because missingness was scarce. A logistic regression was performed for each EPIC domain to study factors related to missingness (Table 7). We first tested the association of each explanatory variable separately (all variables in Tables 1, 2 and 3) and used a liberal *p* < 0.2 threshold of statistical significance for this exploratory stage. Multivariable logistic regression models were then performed including only those explanatory variables that showed potential for association (*p* < 0.2) in the exploratory stage. The threshold of statistical significance in the multivariable models was *p* < 0.05. Sample sizes varied between the five models since there was some missingness also within the explanatory variables.

**Table 1:** Summary statistics of patient characteristics: continuous variables (*n* = 625)

Variables	Mean (SD)	Md (Q_1_, Q_3_)
**Age, yrs**	70.9 (8.8)	71.0 (65.0, 77.0)
**Self-rated health (scale 0–100)**^a^	56.9 (17.6)	56.3 (45.0, 68.8)

^a^ Higher values indicate better self-rated health

**Table 2:** Summary statistics of patient characteristics: categorical variables (*n* = 625)

Variables	Count (%)
**Primary treatment**	
Radiation	260 (41.6)
Prostatectomy	128 (20.5)
Surveillance	155 (24.8)
Hormonal treatment	73 (11.7)
Chemotherapy	9 (1.4)
**EAU cancer risk classification**	
Low risk disease	111 (17.8)
Intermediate risk disease	213 (34.1)
High risk disease	231 (37.0)
Advanced disease	44 (7.0)
Missing	26 (4.2)
**Relationship status**	
In a relationship	526 (84.2)
Not in a relationship	93 (14.9)
Missing	6 (1.0)
**Education level**	
No vocational education	86 (13.8)
Short vocational education	128 (20.5)
Vocational education	156 (25.0)
Polytechnic	157 (25.1)
University education	91 (14.6)
Missing	7 (1.1)
**Working life status**	
Working full-time or part-time	118 (18.9)
Retired	475 (76.0)
Unemployed	11 (1.8)
Outside labour force for another reason	4 (0.6)
Missing	17 (2.7)
**Treatment satisfaction** (at 12 months)	
Extremely satisfied	186 (29.8)
Satisfied	307 (49.1)
Uncertain	60 (9.6)
Dissatisfied	21 (3.4)
Extremely dissatisfied	9 (1.4)
Missing	42 (6.7)

**Table 3:** EPIC scores at 0, 6, and 12 months

EPIC domain	0 months, *n* = 625	6 months, *n* = 598	12 months, *n* = 588	Friedman
	Md (Q_1_,Q_3_)	Md (Q_1_,Q_3_)	Md (Q_1_,Q_3_)	p
Urinary incontinence	100 (79, 100) ^a^	92 (71, 100)	92 (73, 100)	< 0.001
Urinary irritative	81 (75, 94)	88 (75, 94)	88 (75, 94)	0.003
Bowel	96 (83, 100)	95 (83, 100)	92 (83, 100)	< 0.001
Sexual	45 (18, 71)	22 (13, 53)	22 (13, 53)	< 0.001
Hormonal	90 (80, 100)	85 (70, 95)	85 (70, 95)	< 0.001

^a^ Higher values indicate better function

We excluded the EPIC score from the model explaining missingness in the same domain; that is, for example, the EPIC bowel score was not in the model explaining missingness in bowel questions (see Table 7). Based on the EPIC-26 scoring instructions, the summary scores could be calculated only for those participants who had zero (urinary domains) or at most one missing item (other domains). The regression model would thus have excluded all the patients with the most missing items. Otherwise, we used EPIC scores from the six months timepoint to predict the odds of missing answers. Accordingly, we chose the same 6-month measurement point for treatment satisfaction and self-rated health that were both stable between 6 and 12 months. Treatment satisfaction was recoded to reduce small categories. Primary treatment and working life status strongly correlated with age, and their association with any of the five outcome variables vanished after adjusting for age, so they were excluded from the models shown in Table 7. Statistical analyses were performed using IBM SPSS Statistics 28.0.1.0 (IBM, Armonk, NY, USA).

## Results

### Sample characteristics

A total of 625 prostate cancer patients had filled the first questionnaire, 598 the second and 588 patients the third, with a drop-out rate of 5,9% (*n* = 37). Tables 1 and 2 show patient characteristics. The men’s mean age at diagnosis time was 71 years (min = 38, max = 100). Self-rated health mean at diagnosis time was 57. Radiation therapy was the most common cancer treatment received. Over half of the patients had either a low or intermediate risk cancer. Most men were in a relationship, whereas education level varied. Most men were retired (76%). Only 5% of the patients were dissatisfied with treatment. Median PSA level was 7.9 at diagnosis time (Tables 1 and 2).

Table 3 shows EPIC scores at 0, 6, and 12 months. Urinary continence and bowel function were high among study participants throughout follow-up (scores > 90). Sexual function scores were the lowest at every measurement point. Score changes between measurement points were statistically significant.

### Quantity of missing data in EPIC-26 domains and differences between measurement points

Table 4 describes the sum of missing answers per EPIC domain at three measurement points. The number of filled questionnaires slightly decreases along the follow-up due to drop out. When taking all EPIC-26 domains into account, most missing answers appeared at diagnosis time (3,9%). When considering each domain separately, the hormonal domain had the highest proportion of missing answers throughout the follow-up. Sexual domain produced the second-most missing answers (4.4%). Urinary incontinence domain had the smallest missingness proportions at all measurement points. The number of missing answers usually decreased with time, except in the hormonal domain, where the highest missingness was at 6 months, and in the sexual domain, where missingness increased again by 12 months.

**Table 4:** Sum of missing answers per EPIC domain at three timepoints

EPIC domain	No. of items in domain	0 months, 625 filled surveys	6 months, 598 filled surveys	12 months, 588 filled surveys	Mean in domain
Urinary incontinence	4	55 (2.2% ^a^ )	43 (1.7%)	19 (0.8%)	1.6%
Urinary irritative	4	91 (3.6%)	82 (3.4%)	71 (3.0%)	3.3%
Bowel	6	100 (2.7%)	83 (2.3%)	58 (1.6%)	2.2%
Sexual	6	197 (5.3%)	130 (3.6%)	156 (4.4%)	4.4%
Hormonal	5	177 (5.7%)	178 (6.0%)	159 (5.4%)	5.7%
**Mean at time-point**		3.9%	3.4%	3.1%	

^a^ Calculated as follows: 55 missing answers/(625 filled surveys × 4 items in the incontinence domain) × 100 = 2.2%

Table 5 presents the proportion of missing answers in individual questions of EPIC-26 at three timepoints. Proportions exceeding 5% have been marked with a bolded font in the table. The questions with the most missing answers were question 13.b. (breast tenderness/ enlargement, mean 8.5%), question number 10. (frequency of erections, mean 6.7%), question 13.a. (hot flashes, mean 5.9%), and question 8.b. (ability to reach orgasm, mean 5.6%). The urinary incontinence and bowel domains had the lowest missing answer proportions (Table 5).

**Table 5:** Proportions of missing answers in EPIC items at 0, 6 and 12 months post-diagnosis

EPIC domain	Item	0 months, *n* = 625	6 months, *n* = 598	12 months, *n* = 588	Mean
Urinary incontinence	1. Over the past 4 weeks, how often have you leaked urine?	1.3%	1.2%	0.3%	0.9%
	2. Which of the following best describes your urinary control during the last 4 weeks?	1.3%	1.2%	0.3%	0.9%
	3. How many pads of adult diapers per day did you usually use to control leakage during the last 4 weeks?	2.2%	1.2%	0.2%	1.2%
	4. a. How big a problem, if any, has dripping or leaking urine been for you buring the last 4 weeks?	4.0%	3.5%	2.4%	3.3%
Urinary irritative	4. b. How big a problem, if any, has pain or burning of urination been for you buring the last 4 weeks?	3.8%	3.7%	3.6%	3.7%
	4. c. How big a problem, if any, has bleeding with urination been for you during the last 4 weeks?	4.3%	**5.3%**	3.9%	4.5%
	4. d. How big a problem, if any, has weak urine stream or incomplete emptying been for you during the last 4 weeks?	3.4%	2.2%	2.4%	2.7%
	4. e. How big a problem, if any, has need to urinate frequently during the day been for you during the last 4 weeks?	3.0%	2.5%	2.2%	2.6%
Not in domain	5. Overall, how big a problem has your urinary function been for you during the last 4 weeks?	1.4%	1.0%	0.7%	1.0%
Bowel	6. a. How big a problem, if any, has urgency to have a bowel movement been for you?	2.1%	2.5%	1.4%	2.0%
	6. b. How big a problem, if any, has increased frequency of bowel movements been for you?	3.2%	2.8%	1.5%	2.5%
	6. c. How big a problem, if any, has losing control of your stools been for you?	3.5%	2.7%	1.9%	2.7%
	6. d. How big a problem, if any, has bloody stools been for you?	3.5%	3.0%	2.6%	3.0%
	6. e. How big a problem, if any, has abdominal/pelvic/rectal pain been for you?	2.9%	2.5%	1.9%	2.4%
	7. Overall, how big a problem have your bowel habits been for you during the last 4 weeks?	0.8%	0.3%	0.7%	0.6%
Sexual	8. a. How would you rate your ability to have an erection during the last 4 weeks?	4.3%	3.2%	3.9%	3.8%
	8. b. How would you rate your ability to reach orgasm (climax) during the last 4 weeks?	**6.4%**	4.3%	**6.0%**	**5.6%**
	9. How would you describe the usual QUALITY of your erections during the last 4 weeks?	3.5%	2.3%	3.2%	3.0%
	10. How would you describe the usual FREQUENCY of your erections during the last 4 weeks?	**8.3%**	**5.7%**	**6.0%**	**6.7%**
	11. Overall, how would you rate your ability to function sexually during the last 4 weeks?	4.6%	3.7%	4.1%	4.1%
	12. Overall, how big a problem has your sexual function or lack of sexual function been for you during the last 4 weeks?	4.3%	2.5%	3.4%	3.4%
Hormonal	13. a. How big a problem during the last 4 weeks, if any, has hot flashes been for you?	**5.9%**	**6.2%**	**5.6%**	**5.9%**
	13. b. How big a problem during the last 4 weeks, if any, has breast tenderness/ enlargement been for you?	**8.6%**	**8.8%**	**8.0%**	**8.5%**
	13. c. How big a problem during the last 4 weeks, if any, has feeling depressed been for you?	4.2%	**5.3%**	4.6%	4.7%
	13. d. How big a problem during the last 4 weeks, if any, has lack of energy been for you?	4.3%	4.8%	4.4%	4.5%
	13. e. How big a problem during the last 4 weeks, if any, has change in body weight been for you?	**5.3%**	4.5%	4.4%	4.7%

Of all participants with three completed survey rounds (*n* = 579), 37% left at least one EPIC question unanswered during their one-year follow-up and among them it was most common to leave only one or two questions unanswered. The number of questions left unanswered per participant varied between 0 and 32. Of the 579 participants, 10% left ten or more questions unanswered during follow-up. Table 6 shows the number of patients who had left at least one question unanswered in the domain at certain timepoint. In total, 23% of 579 patients skipped at least one question at diagnosis, 19% at 6 months, and 19% at 12 months postdiagnosis. Sexual domain was the only domain in which the number of patients skipping questions changed statistically significantly during the follow-up (Cochran Q = 6.545, p = 0.038). Patients skipped sexual questions the most at diagnosis time (Table 6).

**Table 6:** Number of patients with one or more missing answers in EPIC domain

EPIC domain	0 months	6 months	12 months	Cochran Q
	*n* = 579 ^a^	*n* = 579	*n* = 579	p
Urinary incontinence	30 (5.2%)	22 (3.8%)	16 (2.8%)	0.061
Urinary irritative	30 (5.2%)	35 (6.0%)	34 (5.9%)	0.772
Bowel	29 (5.0%)	24 (4.1%)	22 (3.8%)	0.492
Sexual	67 (11.6%)	49 (8.5%)	49 (8.5%)	0.038
Hormonal	51 (8.8%)	56 (9.7%)	56 (9.7%)	0.783
EPIC total	142 (22.7%)	121 (19.4%)	117 (18.7%)	

^a^ Patients with less than three completed survey rounds are excluded from the table

### Sociodemographic and clinical factors associated to missingness

Odds of missing questions were higher in older men and men with lower education level and poorer self-rated health in all EPIC-26 domains in the univariate analyses (Table 7, *p* < 0.2). Severity of cancer, EPIC scores, and treatment satisfaction had associations to missingness in several EPIC domains. Relationship status was associated to missingness in urinary irritative, sexual, and hormonal domains: Men without relationships had higher odds of missing questions, increasing by 51% in urinary irritative domain and by 97% in the hormonal domain. Men without relationship had 2.7 times higher odds of missing sexual questions compared to men with relationships. However, age was the only factor that remained significant in all domains in multivariable models (*p* < 0.05). Also, treatment satisfaction remained significant in urinary irritative domain, urinary irritative symptoms in the bowel domain, and relationship status in the sexual domain. Education level and bowel symptoms remained significant in the hormonal domain (Table 7).

**Table 7:** Logistic regression models explaining factors related to missingness in each EPIC-26 domain

	Urinary incontinence			Urinary irritative			Bowel			Sexual			Hormonal		
Multivariable model sample size	*n* = 533			*n* = 531			*n* = 530			*n* = 534			*n* = 479		
	OR	95% CI	p	OR	95% CI	p	OR	95% CI	p	OR	95% CI	p	OR	95% CI	p
**Age**	1.087	1.048–1.128	< 0.001*	1.069	1.038–1.101	< 0.001*	1.103	1.063–1.144	< 0.001*	1.075	1.047–0.105	< 0.001*	1.095	1.065–1.127	< 0.001*
**EAU cancer risk classification**			**0.085**			**0.146**			0.293			**0.046**			**0.120**
Low risk disease	1.000			1.000						1.000			1.000		
Intermediate risk disease	0.624	0.250–1.558	0.312	0.963	0.456–2.033	0.992				0.992	0.522–1.886	0.980	0.618	0.333–1.145	0.126
High risk disease	1.548	0.700–3.424	0.280	1.629	0.811–3.274	0.170				1.846	1.011–3.372	0.046	1.068	0.604–1.890	0.821
Advanced disease	1.616	0.505–5.169	0.418	2.067	0.772–5.533	0.148				1.169	0.443–3.086	0.753	1.347	0.569–3.189	0.498
**EPIC scores** ^a^															
Urinary incontinence						0.603			0.272			0.632			0.321
Urinary irritative			0.578				1.018	0.994–1.042	0.138*			0.703	0.986	0.973–0.999	**0.040**
Bowel			0.670			0.354						0.230	0.974	0.961–0.988	< 0.001*
Sexual			0.809			0.316			0.227				0.993	0.984–1.002	**0.113**
Hormonal			0.912			0.517			0.487			0.867			
**Relationship status**			0.747			**0.178**			0.202			< 0.001*			**0.011**
In a relationship				1.000						1.000			1.000		
Not in a relationship				1.514	0.828–2.769	0.178				2.652	1.604–4.386	< 0.001	1.971	1.170–3.320	0.011
**Education level**			**0.027**			**0.060**			**0.006**			**0.006**			< 0.001*
No vocational education	1.000			1.000			1.000			1.000			1.000		
Short vocational education	0.852	0.369–1.969	0.708	1.027	0.500–2.112	0.941	0.678	0.316–1.454	0.318	0.730	0.386–1.380	0.333	1.072	0.583–1.973	0.882
Vocational education	0.785	0.347–1.773	0.560	0.540	0.253–1.153	0.111	0.334	0.145–0.769	0.010	0.473	0.249–0.901	0.023	0.289	0.145–0.575	< 0.001
Polytechnic	0.208	0.069–0.623	0.005	0.428	0.194–0.942	0.035	0.265	0.110–0.639	0.003	0.322	0.162–0.637	0.001	0.303	0.154–0.597	< 0.001
University education	0.365	0.121–1.103	0.074	0.535	0.225–1.275	0.158	0.251	0.087–0.728	0.011	0.386	0.180–0.827	0.014	0.153	0.059–0.399	< 0.001
**Self-rated health** ^a^	0.989	0.974–1.005	**0.163**	0.991	0.978–1.004	**0.155**	0.990	0.975–1.005	**0.174**	0.989	0.978–1.000	**0.048**	0.989	0.978–1.001	**0.068**
**Treatment satisfaction**			**0.121**			0.052*			0.224			0.620			**0.035**
Unsatisfied or extremely unsatisfied	1.333	0.450–3.952	0.604	1.584	0.668–3.758	0.296							0.672	0.307–1.472	0.321
Uncertain	2.267	1.032–4.979	0.042	2.225	1.127–4.391	0.021							1.480	0.577–3.801	0.415
Satisfied or extremely satisfied	1.000			1.000									1.000		

^*^ remained statistically significant (*p* < 0.05) in multivariable model

^a^ higher values indicate better function or self-rated health

## Discussion

EPIC-26 is a commonly used measurement scale used for research purposes and clinical evaluation globally, so it is crucially important to examine the scale’s level and patterns of missing data. We aimed to investigate the prevalence of missing data in different domains of EPIC-26 and to examine potential factors associated with missingness in a sample of 625 Finnish prostate cancer patients.

Missing answer rates in EPIC-26 were low in our Finnish data (3.1–3.9%) between the three measurement points of the follow-up. The hormonal domain produced the most missing answers. Missingness was minimal in the urinary incontinence, irritative, and bowel domains. Missingness did not vary between timepoints except in the sexual domain, where skipping was significantly more common at diagnosis. As much as 63% of patients did not leave any questions unanswered during their personal one-year follow-up, and those who skipped usually skipped only one or two questions. This indicates that EPIC-26 is reasonably easy to answer for many prostate cancer patients.

However, four individual items of EPIC-26 were notable regarding missingness. Questions 13.b. (breast tenderness/ enlargement), 10. (frequency of erections), 13.a. (hot flashes), and 8.b. (ability to reach orgasm) were the most skipped. Questions about breast tenderness and hot flashes might be considered female-specific symptoms and difficult to comprehend unless patients had already experienced gynecomastia and hot flashes from androgen deprivation therapy [13]. Questions about erection frequency and ability to reach orgasm might be difficult to answer if the patient has been sexually inactive. Previous studies have reported that sexually inactive patients might either skip these questions or answer inconsistently [13, 18]. Answering to these most-skipped sexual and hormonal questions can also feel embarrassing, especially, if a person is suffering from these symptoms (low true score), which might lead to data missing not at random (MNAR). Motivation to answer these questions may also be declined because a not-applicable category is missing from the items.

Our previous study utilizing qualitative data from the same study population found that patients with a suprapubic urinary catheter could not find a suitable answer option for urinary function questions [18]. However, in the statistical data this issue did not appear as conspicuous non-response in the urinary questions. In the same study we reported that men using an erectile aid struggled to answer sexual function questions. Missing answer rates were clearly higher in the sexual domain than in the urinary or bowel domains, which could be an indication of confusion related to lack of instructions related to erectile aid use or sexual inactivity, or the aforementioned unwillingness to report low sexual function.

We found several factors associated with missingness in different questionnaire domains, indicating the possible presence of MAR data. Higher age, lower education level, no relationship, more severe cancer, lower function scores in some EPIC domains, lower treatment satisfaction or self-rated health were significant predictors in the univariate models. However, only a few associations remained above the threshold of statistical significance in the multivariable regression models due to our data’s low missing answer rates. Similar connections have been found with a depression scale (SDS) and a dietary instrument (MedDietScore) where higher missingness rate was significantly associated with older age, lower educational level, and poorer health status [4].

Our results yield an important insight into difficulties in answering: Older men with a lower education level and possibly poorer health status might require assistance to complete EPIC-26. This is especially important at the time of diagnosis when prostate cancer patients are asked to complete the first questionnaire under stressful circumstances. A new finding in our study was the importance of a relationship in completing surveys: It is known that especially female partners might take responsibility on their spouses’ health [22] and thus might assist prostate cancer patients in answering.

Because EPIC summary scores can be calculated only for those participants who have zero (urinary domains) or at most one missing item (other domains), and patients with poorer health status tend to skip more EPIC questions than others, missingness can result in excessively positive EPIC average scores in a study population. We consider it important for researchers to report the proportion of missing answers in their articles and to check for possible patterns of missingness in their data.

## Conclusions

The missingness level in EPIC-26 in a Finnish study population was generally acceptable. However, with the four most-skipped questions in the sexual and hormonal domains of EPIC-26, the questions themselves or their underlying assumptions may lead to missing responses. This means that these responses are not missing at random (MNAR) and thus require careful consideration [5]. To improve validity of the measure, we very much agree with Lee et al. (2018) [13] that the questionnaire’s hormonal section requires an additional explanation that the inquired symptoms are common treatment side effects of anti-androgen therapy. Furthermore, as previously suggested, questions 8–10 require either a not-applicable category for sexually non-active patients [18] or at least an additional instruction, “Please try your best to answer the following questions even if you do not have any sexual activity” [13]. These minor modifications would decrease the level of missing answers in EPIC measures.

### Limitations

The number of missing answers in the study population was low, so we were forced to combine information from the whole follow-up period instead of studying each timepoint separately in the regression models. With the low missingness levels in our survey, we probably did not observe all existing connections between the explanatory variables and missingness in the different EPIC-26 domains. We found most associations in the domains with the most missingness. The logistic regression model also excluded more patients from the model with every added explanatory variable because there was also some missing information within the explanatory variables. Low true score in explanatory variables or high age may have also led to missingness in the explanatory variables themselves. We suppose that the observed associations between explanatory variables and missingness in EPIC domains could have been stronger if there were no missingness within the explanatory variables themselves. We were still able to find several significant associations with univariate models that offer insight into factors that might hinder answering. Our results can be conceptualized as an exploratory effort in this domain, and it would be useful to explore the level of inconsistent answers in EPIC measures.

### Supplementary Information

**Additional file 1.** EPIC-26 The Expanded Prostate Cancer Index Composite Short Form.

## Data Availability

The datasets analyzed during the current study are not publicly available due to restrictions in research permission but are available from the corresponding author on reasonable request.
